# Very Late Extramedullary Relapse of Acute Myeloid Leukemia 13 Years After Allogeneic Transplant: A Case Report

**DOI:** 10.1155/crh/8840484

**Published:** 2026-06-02

**Authors:** Yvette von Aarburg, Astrid Beerlage, Darius Juskevicius, Cyrill Rütsche, Jan Dirks, Rainer Grobholz, Britta Hartmann, Roby Mathew, Katharina Leonards, Sabine Gerull

**Affiliations:** ^1^ Department of Hematology and Oncology, Kantonsspital Aarau AG, Aarau, Aargau, Switzerland; ^2^ Department of Hematology, Universitatsspital Basel, Basel-Stadt, Basel, Switzerland; ^3^ Department of Laboratory Medicine, Diagnostic Hematology, Basel University Hospital, Basel, Switzerland, ukbb.ch; ^4^ Hematologic Laboratory, Kantonsspital Münsterlingen, Münsterlingen, Thurgau, Switzerland; ^5^ Institute of Pathology, Kantonsspital Aarau AG, Aarau, Switzerland, klinikum-fuerth.de; ^6^ Medical Faculty, University of Zurich, Zurich, Switzerland, uzh.ch; ^7^ Department of Medical Genetics, Institute of Laboratory Medicine, Kantonsspital Aarau AG, Aarau, Aargau, Switzerland; ^8^ Molecular Diagnostics, Laboratoire National De Santé, National Center of Genetics, Luxembourg District, Dudelange, Luxembourg

**Keywords:** acute myeloid leukemia, extramedullary relapse, HNRNPK mutation, late relapse, testicular myelosarcoma

## Abstract

Relapse of acute myeloid leukemia (AML) typically occurs within the first 3 years following treatment, with very late relapses being rare and often presenting extramedullary. We report a unique case of extramedullary AML relapse occurring 13 years after allogeneic hematopoietic stem cell transplantation. The patient developed a myelosarcoma, prompting molecular and histological investigation to determine the origin and mutational landscape of the disease. Next‐generation sequencing (NGS) was performed on archived bone marrow samples from the initial AML diagnosis in 2007 and on the relapsed tumor tissue from 2021. The 2007 sample contained nine somatic variants, while the 2021 myelosarcoma harbored four variants. Only a single somatic mutation, a frameshift alteration in the *HNRNPK* gene, located on 9q21, was shared across both timepoints, suggesting a potential founder event and supporting the hypothesis of clonal evolution. Chimerism analysis and histopathological findings confirmed that the relapse originated from the patient rather than from donor‐derived hematopoiesis. This case underscores the importance of long‐term vigilance in AML survivors, especially in those receiving chronic immunosuppression, as impaired immune surveillance may allow for reemergence of malignant clones. The persistent *HNRNPK* mutation across both disease phases may point to a rare but critical driver of leukemogenesis and relapse. Our findings highlight the utility of comprehensive molecular profiling in elucidating disease dynamics and informing personalized monitoring strategies.

## 1. Introduction

Relapse in acute myeloid leukemia (AML) occurs in about half of patients after initial remission, with most relapses happening within the first 3 years, though late relapses, which often occur at extramedullary sites, tend to have a better prognosis [1, 2]. Next‐generation sequencing (NGS) plays a key role in identifying genetic alterations in AML, revealing that different subclones can evolve over time, causing the mutational pattern at relapse to differ from that at diagnosis. This report details a rare case of very late extramedullary relapse of secondary AML in the testicle, focusing on the changes in mutational patterns between initial diagnosis and relapse.

## 2. Case Report

In 2007, a 45‐year‐old man presented with intermittent fever, night sweats, shivering, headaches, and limb pain. The blood count revealed normocytic, normochromic anemia with 3.4% of myeloid blasts. Bone marrow biopsy confirmed myelodysplastic syndrome (MDS), specifically refractory anemia with excess blasts II (WHO Classification, Third Edition, 2001) [3], which quickly progressed to AML with 53% peripheral blasts, normal karyotype (46, XY), and no detectable *FLT3* mutation. Molecular analyses beyond *FLT3* were not performed at that time. The patient had no family history of hematologic malignancies or hereditary cancer syndromes. His psychosocial history was unremarkable, with no notable exposures or substance use. Physical examination was normal, with no identifiable focus of infection.

The patient underwent two cycles of induction chemotherapy (on the HOVON42/SAKK30/00 study) and consolidation with autologous hematopoietic stem cell transplant (HSCT) following conditioning with busulfan and cyclophosphamide. Early progression led to reinduction chemotherapy with cytarabine and idarubicin and due to disease persistence to salvage therapy with FLAG‐Ida (fludarabine, cytarabine, granulocyte‐colony stimulating factor, idarubicin), resulting in complete remission (CR). He then underwent myeloablative allogeneic HSCT from an unrelated donor, with an HLA‐C and HLA‐DRB4 mismatch, achieving 100% donor chimerism. Post‐transplant, he developed chronic graft‐versus‐host disease (cGvHD) affecting multiple organs, namely, severe sicca syndrome of the eyes and enoral mucosa with leucoplakias of the tongue, requiring long‐term immunosuppression.

Likely due to chronic immunosuppression combined with cGvHD, the patient developed recurrent squamous cell carcinomas of the tongue, necessitating multiple partial resections. Bone marrow biopsies showed ongoing remission, and short tandem repeat (STR)–based chimerism analysis confirmed 100% donor chimerism.

In 2021, 13 years post‐transplant, the now 59‐year‐old patient presented with a unilateral testicular mass. Histology from orchiectomy revealed myeloid blast infiltration, indicating myelosarcoma. Comprehensive staging, including PET‐CT and bone marrow biopsy, found no other disease manifestation, suggesting a solitary extramedullary relapse of AML. Bridging therapy with azacitidine and venetoclax was initiated, and a second allogeneic HSCT was planned.

After three cycles, PET‐CT confirmed CR, and the patient underwent conditioning with fludarabine/busulfan (2 days) followed by HSCT from a new unrelated donor with an HLA‐DPB1 mismatch (*Cytomegalovirus* [CMV] D/R pos/neg; Epstein–Barr virus [EBV] D/R pos/pos). One month post‐transplant, 100% chimerism from the second donor was achieved. The patient received a prophylactic irradiation of the contralateral testicle with 30 Gray.

Post‐transplant complications included CMV colitis and continuing chronic ocular and oral GvHD leading to another squamous cell carcinoma relapse, requiring a complete glossectomy.

**FIGURE 1:**
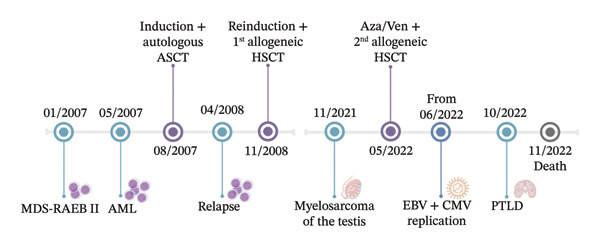
Time course of the patient’s medical history, highlighting key events and interventions over the specified period. MDS‐RAEB II = myelodysplastic syndrome with refractory anemia and excess blasts II, AML = acute myeloid leukemia, ASCT = autologous stem cell transplant, allo‐HSCT = allogeneic hematopoietic stem cell transplant, EBV = Epstein–Barr virus, CMV = *Cytomegalovirus*, PTLD = post‐transplant lymphoproliferative disorder, and Aza/Ven = azacitidine/venetoclax.

Six weeks post‐transplant, asymptomatic EBV replication was treated with rituximab. However, by October 2022, the patient developed EBV‐associated post‐transplant lymphoproliferative disorder (PTLD) in the brain, leading to rapid deterioration and death from respiratory failure due to brainstem lymphoma infiltration (Figure 1).

To understand the leukemia’s origin, we conducted chimerism analyses (based on STRs using Promega GenePrint 24) on DNA samples obtained from both blood and myelosarcoma. The peripheral blood displayed 100% donor chimerism, while the myelosarcoma exhibited mixed chimerism with 38% donor cells (data not shown).

Histological examination of the testicle revealed a lymphocyte infiltration of approximately 20%, presumed to be of donor origin given the 100% chimerism in the blood. Considering the inherent variability in visually estimating lymphocyte infiltration and correlating to STR‐based chimerism, we inferred that the donor chimerism was attributed to the lymphocyte infiltration. Consequently, we deduced that the remaining assessed cells, including the testicular tissue and, notably, the myeloblasts, originated from the recipient.

To further differentiate between patient‐ and donor‐derived leukemia and to investigate genomic evolution, we conducted a retrospective NGS analysis of 523 genes using the Illumina TruSight Oncology 500 protocol and tertiary analysis using Qiagen Clinical Insight on the myelosarcoma and material from 2007.

**FIGURE 2:**
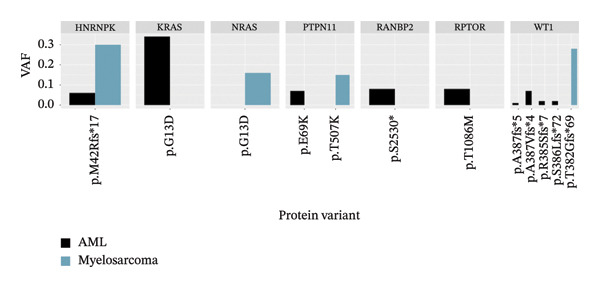
Variant allelic frequency (VAF) of acute myeloid leukemia (AML) (2007) and myelosarcoma (2021), measured using TruSight Oncology 500 High‐Throughput (TSO500) sequencing. The graph illustrates the distribution of VAF values for both cancer types, highlighting differences and similarities in mutational profiles between the AML 2007 and myelosarcoma 2021 samples. The figure was generated using rtutor.ai (https://github.com/gexijin/RTutor).

At diagnosis in 2007, NGS identified 9 presumably somatic variants in *HNRNPK*, *KRAS*, *PTPN11*, *RANBP2*, *RPTOR*, and *WT1*. The myelosarcoma harbored 4 presumably somatic variants in *HNRNPK*, *NRAS*, and different *PTPN11* and *WT1* variants, with only the *HNRNPK* mutation remaining consistent between the primary AML and the testicular relapse (Figure 2).

The p.M42fs∗17 mutation in the *HNRNPK* gene results in a frameshift, leading to underexpression of hnRNP K, a protein involved in mRNA splicing and stability. This rare somatic mutation, absent from germline databases, is predicted to cause nonsense‐mediated mRNA decay, and loss‐of‐function in *HNRNPK* has been linked to leukemogenesis, especially in AML with 9q deletions [4–7]. Dysregulation of hnRNP K contributes to tumorigenesis, with *HNRNPK* haploinsufficiency leading to defects in key regulators like C/EBPα and p21 [8, 9]. In this patient, the mutation likely played a pivotal role in leukemia development, suggesting that the myelosarcoma represented a very late relapse of the original AML rather than a new or donor‐derived leukemia. It remains unclear why similar yet different mutations (*WT1, PTPN11, KRAS,* and *NRAS*) appeared in the primary AML and at relapse. Most likely, the environment was particularly permissive for RAS pathway activation, such that leukemic cells acquiring mutations in RAS pathway–associated genes gained a strong selective advantage, enabling them to outcompete other subclones.

## 3. Discussion

Relapses in AML typically occur early, often displaying clonal evolution [10]. Ding et al. explored genetic changes linked to AML relapse by performing whole genome sequencing (WGS) on primary tumor–relapse pairs from 8 patients. Their study demonstrated clonal evolution in all cases, revealing common, new, or lost mutations between diagnosis and relapse suggesting that chemotherapy may influence the mutation spectrum, potentially contributing to relapse [11]. Yilmaz et al. also examined late AML relapse cases by performing whole exome sequencing of 10 patients. In eight cases, the founding clone from the primary tumor evolved into the relapse clone, gaining new mutations. In two cases, the relapse clone differed from the founding clone. Some subclones from the primary tumor were eradicated by chemotherapy and not detected at relapse. Late relapses are rare but often involve the persistence of the founding clone with additional mutations [12]. Both studies suggest that chemotherapy alone, even without transplantation, exerts selective pressure that promotes therapy‐resistant subclones through clonal evolution.

Both the initial and relapsed AML in our case involved mutations in RAS pathway genes (*KRAS*, *NRAS*, and *PTPN11*) and *WT1*, with a shared mutation in *HNRNPK*, which is linked to leukemia.

**FIGURE 3:**
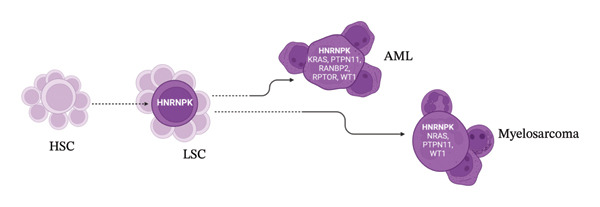
Evolution of the hematopoietic stem cell (HSC) into a common leukemic stem cell (LSC) into two different diseases: acute myeloid leukemia (AML) (2007) and myelosarcoma (2021).

We hypothesize that an early leukemic stem cell (LSC) clone with these mutations existed before the initial diagnosis and evolved under the pressure of different therapeutic regimes, leading to relapse with new mutations. The recurrence of the identical HNRNPK p.M42Rfs∗17 variant supports the persistence of a small population of preleukemic or treatment‐resistant hematopoietic stem/progenitor cells that survived initial therapy and later expanded into a genetically distinct secondary malignancy. Given the tumor‐suppressor role of HNRNPK, successive lines of therapy may have facilitated clonal selection and the emergence of cooperating driver mutations. Notably, the relapse occurred in the testis, an immune‐privileged site, suggesting the LSCs avoided immune detection possibly due to cGvHD and long‐term immunosuppression (Figure 3).

Testicular relapse highlights the challenge of AML recurrence in immune‐privileged sites, similar to the central nervous system. These sites can evade immune surveillance, allowing dormant leukemic cells to persist [13]. Prolonged immunosuppression due to medication and cGvHD likely weakened immune responses, facilitating this relapse. This case is an example of rare late relapse in an extramedullary site, which may differ from bone marrow relapses. A series of cases from Watts et al. with very late relapses (over 10 years) also showed recurrence in extramedullary sites, suggesting a different mechanism associated with impaired immune surveillance [14]. Additionally, Kikushige et al. reported a case of very late relapse (11 years post‐transplant) and as well EBV‐associated PTLD, suggesting impaired immune surveillance due to prolonged immunosuppression [15]. Our patient also had EBV‐associated PTLD, CMV reactivation, and recurrent SCC of the tongue.

A major strength of this case is the availability of archived material from 2007, which allowed direct comparison between the initial AML and the extramedullary relapse using retrospective NGS and STR analyses. This helped confirm a shared founder clone. The study also has limitations. The retrospective design restricted the analyses we could perform, and some variants cannot be clearly classified as driver or passenger. Use of a TSO500 panel may have missed additional mutations or cytogenetic abnormalities present at diagnosis, reducing the completeness of clonal reconstruction, and the lack of intermediate samples between 2007 and 2021 limits the precision of the inferred evolutionary timeline.

In addition, although the patient did not exhibit clinical features typically associated with Au–Kline syndrome (AUKS), a multisystem neurodevelopmental disorder caused by heterozygous germline loss‐of‐function variants in HNRNPK, the possibility of a constitutional or rare mosaic HNRNPK defect cannot be fully excluded. AUKS is characterized by hypotonia, developmental delay, markedly delayed or absent speech, distinctive craniofacial features, and occasional congenital anomalies involving the heart, kidneys, skeleton, craniofacial structures, and autonomic nervous system [16]. Because pathogenic AUKS‐associated variants are typically loss‐of‐function alterations, the repeated detection of the HNRNPK p.M42Rfs∗17 frameshift mutation across independent malignancies necessitates consideration of a germline or mosaic origin. Confirmation would ideally require sequencing of nonhematopoietic tissue or, alternatively, blood obtained during remission prior to allogeneic transplantation; however, no such material was available, and therefore a germline origin cannot be conclusively ruled out. Likewise, sequencing of donor‐derived material could have provided valuable complementary information, but NGS of donor blood was not feasible because no donor samples were accessible for retrospective analysis.

Taken together, this case underscores the risk of late AML relapse in extramedullary sites and highlights the need for vigilant long‐term monitoring, especially in patients with cGvHD and extended immunosuppression.

## Author Contributions

S.G., Y.A., and K.L. planned the project. K.L., C.R., J.D., R.G., R.M., and B.H. performed the analyses.

S.G., Y.A., K.L., A.B., and D.J. analyzed the data and wrote the manuscript.

## Funding

No external funding was received.

## Consent

The patient gave his oral consent to the planned research but died prior to manuscript completion.

## Conflicts of Interest

The authors declare no conflicts of interest.

## Data Availability

The data that support the findings of this study are available on request from the corresponding author. The data are not publicly available due to privacy or ethical restrictions.
